# Interdigitated Electrode for Electrical Characterization of Commercial Pseudo-Binary Biodiesel–Diesel Blends

**DOI:** 10.3390/s21217288

**Published:** 2021-11-01

**Authors:** Inocêncio Sanches dos Santos-Neto, Christian Diniz Carvalho, Gilberto Balby Araújo Filho, Cassio Daniel Salomão Silva Andrade, Giselle Cutrim de Oliveira Santos, Allan Kardec Barros, João Viana da Fonseca Neto, Vicente Leonardo Paucar Casas, Luciana Magalhães Rebelo Alencar, Alberto Jorge Oliveira Lopes, Fernando Carvalho Silva, Francisco Sávio Mendes Sinfrônio

**Affiliations:** 1 Department of Electrical Engineering, Campus Bacanga, Federal University of Maranhão, São Luís 65080-805, Brazil; christiandiniz@gmail.com (C.D.C.); gbalby26@gmail.com (G.B.A.F.); cassiodaniel@outlook.com (C.D.S.S.A.); akduailibe@gmail.com (A.K.B.); vianafonseca@gmail.com (J.V.d.F.N.); Lpaucar@ieee.org (V.L.P.C.); kjvida@mac.com (F.S.M.S.); 2 Biological and Health Sciences Center, Campus of Bacanga, Federal University of Maranhão, São Luís 65080-805, Brazil; giselle.cutrim@gmail.com (G.C.d.O.S.); lopesajo@gmail.com (A.J.O.L.); 3 Department of Physics, Campus of Bacanga, Federal University of Maranhão, São Luís 65080-805, Brazil; lucianamagal@gmail.com; 4 Department of Chemistry, Campus of Bacanga, Federal University of Maranhão, São Luís 65080-805, Brazil; fcs.ufma@gmail.com

**Keywords:** interdigitated electrodes, pseudo-binary biodiesel–diesel blends, capacitive sensor

## Abstract

Non-standard diesel blends can be harmful to the environment and human health. In this context, a simple analytical method to estimate the biodiesel mixture ratio in diesel was developed based on impedance spectroscopy (IS) associated with interdigitated sensors. In this article, four different interdigitated sensors with varied comb spacing (G) were simulated using the COMSOL Multiphysics software. Based on finite element simulations, four interdigitated electrode architectures were manufactured and evaluated. The best geometry was chosen according to theoretical data simulations, and its interdigitated electrodes were manufactured for the compositional evaluation of pseudo-binary biodiesel–diesel mixtures. According to the X-ray powder diffraction technique, the deposition of the conductive layer (Au^0^) over the surface of the dielectric substrate (SiO_2_) did not alter its phase composition. In the analysis of AFM and SEM, it was possible to observe irregular edges on the electrodes, possibly related to the manufacturing process of the thin layers and mechanical stability. Another characteristic observed in the AFM images was the height of the step of the gold layer of the sensor. Several cross sections were obtained, and the mean step value was 225.71 ± 0.0032 nm. Although there were differences in the roughness, the whole sensor had nanometric roughness. Based on the finite element method simulation performed, it can be assumed that the geometric parameters more suitable for the manufacturing of the electrode are W = 20 µm, L = 1000 µm, G = 50 µm, and N = 40 digits. The electrical characterization performed by impedance spectroscopy showed that we could differentiate between biodiesel and diesel fuels and their pseudo-binary mixtures in the low-frequency region.

## 1. Introduction

By definition, biodiesel is a complex mixture of long-chain esters, often produced by the transesterification of vegetable oils and/or animal fats using short-chain alcohol and homogeneous or heterogeneous catalysts [1]. Due to its combustion properties and being a fossil-based combustible, it can be used as a fuel itself or as an additive for commercial diesel blends in varying proportions.

In Brazil, the National Agency of Petroleum, Natural Gas and Biofuels (ANP) is responsible for, among other things, controlling the local commercialization of fuels and regulating the volume of additives added to liquid fuels, such as in biodiesel–diesel pseudo-binary blends. To do so, the ANP currently recommends that all commercial diesel blends must be evaluated according to EN14078, ASTMD7371, and NBR15568 standard methodologies using near-infrared spectroscopy and multivariate analysis [2]. Despite their extremely high operational costs, several authors have suggested the development of alternative methodologies based on chromatography analyses or nuclear magnetic resonance spectroscopy [3,4,5].

Electrochemical impedance spectroscopy (EIS) is a powerful experimental technique that compares the electrical response of a system to a time varying electrical excitation [6]. Recently, electrochemical impedance spectroscopy using interdigitated sensors emerged as an affordable technology for evaluating the dielectric properties and chemical composition of pseudo-binary fuel blends [7,8]. González Prieto et al., for instance, used impedance spectroscopy for the dielectric characterization of diesel and biodiesel samples, reporting room-temperature dielectric constants of 2.2 and 3.3, respectively [9]. Similar results were reported by [10].

In this context, [11,12] have suggested that interdigitated electrodes can be used in association with impedance spectroscopy to predict the dielectric performance of liquid fuel samples and, at a given level, correlate them to their chemical composition.

Currently, interdigitated electrodes have been used in surface acoustic wave devices [13], acoustic transducers [14], and tunable devices [15]. They have also been used as probes for dielectric spectroscopy [16] due to their design flexibility, reduced sizes, and affordable prices, being able to detect multiple physical properties in different frequency domains [17]. Apart from these, interdigitated electrodes have also been broadly utilized in biological and chemical sensing, in which the changes in capacitance or impedance have been gauged based on the relationship between the analyte and a sensitive layer [18].

The advantages of these devices are the miniaturization of the electrodes, low manufacturing cost, and the potential for use in a series of applications without substantial changes in the sensor design. Moreover, interdigitated electrodes present promising advantages in terms of low ohmic drop, the fast establishment of steady-state, rapid reaction kinetics, and an increased signal-to-noise ratio [19]. The structure includes two parallel coplanar electrodes, with periodical repetition of design (length, width, and electrode spacing).

The use of interdigitated electrodes eliminates the need for a reference electrode and provides a simple means for obtaining a steady-state current response. Since anodic and cathodic electrodes are put together, minute amounts of species can be evaluated between the electrodes [20].

It is noteworthy that in works described in the literature, the application of parallel plate electrodes (electrodes in carbon paste, and stainless-steel electrodes) associated with impedance spectroscopy to determine the non-conformity of these biodiesel and diesel blends can be observed. However, the interdigitated electrodes developed in this work are of low manufacturing cost and have the potential for use in a series of applications without substantial changes in the sensor design.

In the present work, due to its versatility, the impedance spectroscopy technique was used to determine additives in biodiesel and diesel mixtures. However, the analyses were carried out without any pre-treatment of the fuel samples. Moreover, this work aims to evaluate the effect of the design and chemical composition of the interdigitated electrode on its sensitivity to pseudo-binary biodiesel–diesel blends.

## 2. Materials and Methods

### 2.1. Interdigitated Electrode Design by the Finite Element Method

Computational simulation offers the possibility of exploring the sensitivity of different geometric configurations of prototypes before their manufacture. In this paper, four distinct interdigital electrodes were designed by varying comb spacing (G) and keeping the digit width (W) and length (L) constant (Table 1).

**Figure 1:**
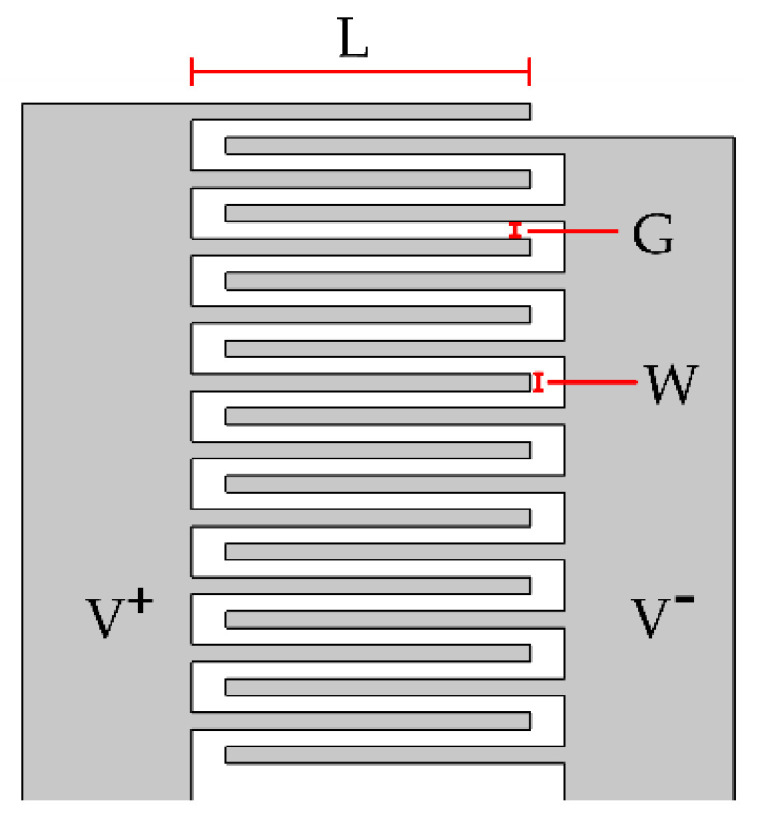
Two-dimensional (2D) schematic representation of the interdigitated electrodes.

Each interdigitated electrode consisted of a group of twenty parallel buses connected alternately to the hot (0.2 V) and ground terminals (Figure 1).

Dielectric studies were performed using the electrostatic interface available on the AC/DC module of the COMSOL Multiphysics platform. In this way, the electric field distribution, electric field magnitude, and the capacitance for each electrode were predicted using a 3D-planar geometric model developed with 231,894 tetrahedral elements and appropriate boundary conditions.

The equations for determining the capacitance in each finite element are

(1) $$-\nabla \varepsilon_{0\;}\varepsilon_{r}\nabla V=\rho$$

(2) $$D=\varepsilon_{0}\varepsilon_{r}E$$

where ρ is the charge density, D is the electric displacement, and E is the electric field. The electrostatic energy density needed to charge a capacitor is equal to the electrostatic field energy and is given by

(3) $$W_{e}=\int_{\Omega }\mathrm{DE}d\Omega$$

Then, capacitance is given by

(4) $$C=\frac{2}{V_{\mathrm{input}}^{2}}\int_{\Omega }W_{e}d\Omega$$

where V_input_ is the voltage applied to the input of the sensor.

After finite element analysis and electrical prospecting, each interdigitated electrode (sensor) was manufactured by a photolithography process using quartz substrates (28.0 mm × 5.0 mm × 0.4 mm). In this process, metallic chrome (Cr^0^_thickness_ = 5.0 nm) and gold (Au^0^_thickness_ = 25.0 nm) layers were deposited by the sputtering technique on the cleaned surfaces of the quartz substrates. The photoengraving of the electrode on the quartz substrate was done by means of photomask designed in an integrated CAD environment.

### 2.2. Chemical and Physical Characterization of the Interdigital Electrode

Wavelength dispersive X-ray fluorescence (WDXRF) spectra were obtained by Tiger S8 (Bruker, Billerica, MA, USA) tube with rhodium (Rh), which operated at 30–50 kV/20–33 mA, with XS-55, PET, LiF200, XS-GE-55 crystals, and 0.23° or 0.40° collimators. Myler^®^ films (3.6 mM) and Teflon recipients were used.

X-ray powder diffraction (XRPD) measurements were recorded using a D8 advance diffractometer (Bruker, Billerica, MA, USA) with a CuKα (λ = 1.5406 Å) tube operating at 40 kV/40 mA and a LynxEye linear detector. The data were collected in Bragg-Bretno geometry, in the range of 20–100°, with a step size of 0.02° and a counting time of 0.5 s. The XRPD patterns were compared with the Joint Committee Powder Diffraction Standards (JCPDS) data for the phase evaluation.

Three-dimensional topographic images and electrode roughness analysis were performed using a Multimode 8 (Bruker, Billerica, MA, USA) atomic force microscope with QNM (peak force tapping quantitative nanomechanics) mode. Nominal spring constant ScanAsyst-Air (Bruker, Billerica, MA, USA) probes of 0.4 N m^−1^ and nominal tip radius of 2 nm were adopted.

A topology evaluation was carried out using a scanning electron microscope Zeiss EVO HD15 (Zeiss, Oberkochen, Germany), with 150 X magnification, a vacuum pressure of 10^−7^ Pa, and an electron acceleration voltage of 5 kV. The compositional analysis of each device was predicted using X-ray dispersive energy spectrometry (EDX) using Quantax/Bruker equipment.

Finally, the electrical properties of the sensors were estimated using a frequency response analyzer Solartron model 1260 (Solartron, Farnborough, UK), in which the designed interdigitated electrodes were connected by shielded cable and BNC inputs, both with 50 Ω impedance. All measurements were carried out using 0.2 V and five points for decades, in a frequency range between 1 and 1 × 10^6^ Hz. The measurements were performed at a temperature of 21.0 ± 0.1 °C. The whole process was computer controlled. To minimize the effects of external interference, a conductive arrangement based on Faraday’s cage principle was used.

## 3. Results

### 3.1. Finite Element Analysis and Electrical Evaluation

Some studies have been developed to increase the capacitance ratio per area of the interdigitated electrodes [21]. As capacitance increases, the sensitivity of a device based on interdigitated electrodes also increases if the analyte remains within the sensitive area. Thus, the device size can be reduced while the sensitivity value is kept constant [22].

**Figure 2:**
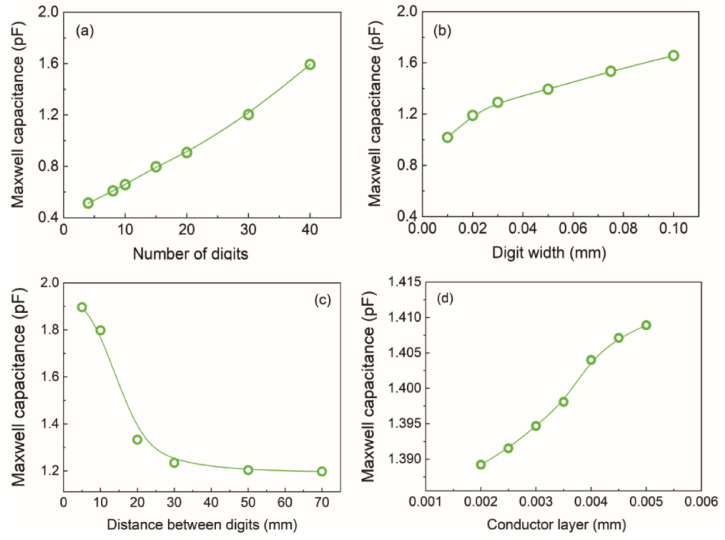
Maxwell capacitance as a function of the (**a**) number of digits, (**b**) digit width, (**c**) distance between digits, and (**d**) conductor layer of the interdigitated electrodes.

According to [23], the sensitivity of any electronic sensor is dependent on its architecture. Thus, this work evaluated the influence of the number of digits, conductive layer thickness, digit width, and the distance between the digits on the Maxwell capacitance of the interdigitated electrodes. As shown in Figure 2, all interdigitated electrodes were assembled as a coupling of adjacent conducting digits where the general capacitance was equivalent to the sum of each capacitor created in each pair of combs. Thus, the increase in the number of conducting digits promoted a linear increase in Maxwell capacitance (Figure 2a). Similarly, the estimated Maxwell capacitance was proportional to the width of the digits in the sensor due to the edge field effect that occurred on the surface of the interdigitated electrodes [24].

According to [25], having wide spacing between interdigitated electrodes produces more uniform electric field lines and a more optimized strain coefficient. However, to produce an equivalent electric field with these dimensions, it would be necessary to apply higher voltages, increasing energy consumption. In contrast, a decrease in electrode spacing will make the manufacturing process more difficult and increase the likelihood of defective conductive layer paths.

According to [26], the digit width parameter must be optimized, since it can lead to electrical field distortions at the conductive track edges. In interdigitated electrodes, the capacitance is given mainly by the electromagnetic interaction between the two electrodes’ conductive track edges. Thus, considering a fixed-size sensor, the greater G and W, the smaller the number of edges in the tracks of the electrodes, which reduces the value of the capacitance and hence its sensitivity. The influence of conductor metal layer thickness on the capacitance of the interdigitated electrodes was determined using the following geometry: W= 20 µm, L = 1000 µm, G = 50 µm, and N = 40 digits. According to [27], the electrode thickness does not influence a parallel plate capacitor’s capacitance. However, in an interdigital capacitor, the electrode thickness has a discernible influence on the device capacitance. As shown in Figure 2d, this electrical property varies similarly to a sigmoid with the conductor layer due to the contribution of the air-filled capacitor.

One of the operating principles of the interdigitated capacitive sensor used in this work is based on parallel plate capacitors. Once an AC voltage is applied between the positive and negative electrodes, electric fields are generated. If there is any material nearby, the generated electric fields penetrate the materials, which leads to changes mainly in the capacitive reactance of the sensor and hence its impedance [28].

**Figure 3:**
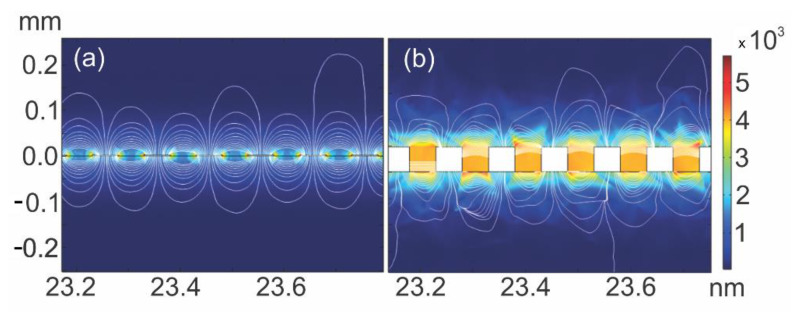
Electric field lines for the interdigitated electrode with (**a**) 0.00254 nm and (**b**) 0.04754 nm.

According to the finite element simulation, the electric field lines generated on the interdigitated electrode surface had a “fringe-like distribution” throughout all the comb pairs (Figure 3a). However, these electrical field lines were widely distorted when the conductive layer was enlarged, probably due to parasitic capacitive phenomena (Figure 3b).

Due to the coplanar nature of the sensor, there were more prominent fringing field lines in addition to the direct field lines between the fingers; this made the coplanar-type capacitive sensor more sensitive to the changes in the electrical properties of the surrounding medium. Therefore, by measuring the changes in the impedance, the electrochemical sensing behavior could be observed [29].

### 3.2. Interdigitated Electrode Description

#### Structural Characterization

**Figure 4:**
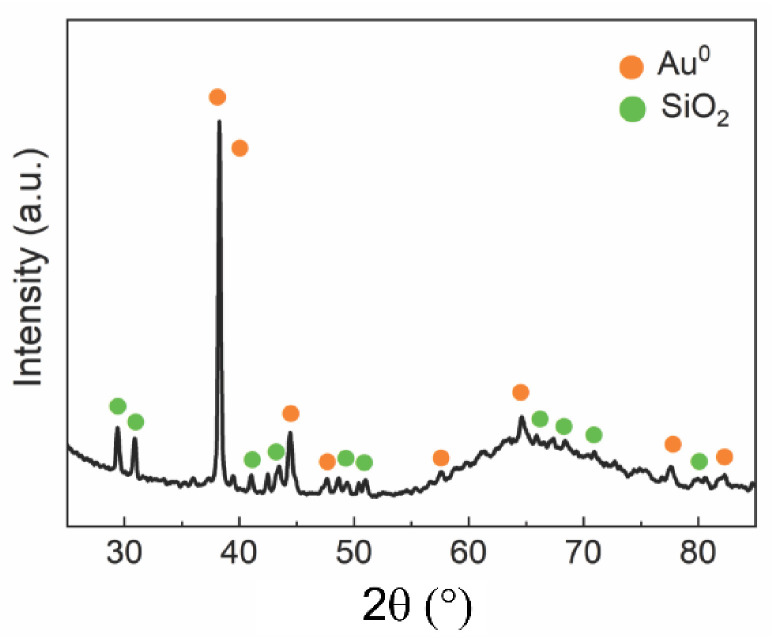
XRD patterns of interdigitated electrodes: Au^0^ (conductor), SiO_2_ (dielectric substrate).

X-ray diffraction analysis was performed to verify the materials used in the manufacture of the electrodes and possible contamination of the interdigitated electrodes by other metals, which could lead to errors in electrical measurements. Figure 4 shows the diffractogram of the interdigitated electrodes and their index peaks.

By comparing the sample diffractograms with the standard JCPDS plugs, diffraction peaks resembling two distinct phases were observed on the electrode surface: Au^0^ (conductor) and SiO_2_ (dielectric substrate). The metallic gold was deposited on the dielectric substrate surface as a cubic system (space group Fm-3m), with crystallographic peaks at 38.28° (111), 44.42° (200), 64.62° (220), 77.71° (311), and 82.34° (222) [30]. In contrast, the quartz dielectric substrate (SiO_2_) was generated as a hexagonal structure (space group P3121), with crystallographic peaks at 29.31° (100), 30.94° (101), 39.51° (102), 41.52° (111), 42.48° (200), 43.48° (201), 50.15° (112), 59,98° (211), 67.76° (212), 68.16° (203), 79.90° (213), and 81.52° (310).

**Figure 5:**
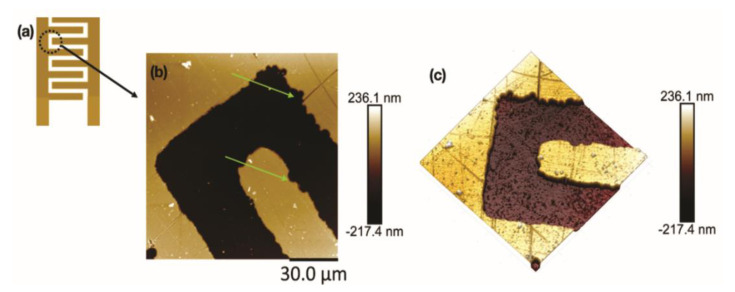
Atomic Force Microscopy topographic images of the interdigital region of the sensor. (**a**) Schematic figure of the sensor architecture. The AFM image was obtained in the region highlighted by the circle. (**b**) AFM height image showing the electrode surface and a digit. The green arrows indicate defects in the deposited layer. (**c**) Three-dimensional image corresponding to the image (**b**). One can observe the grooves on the electrode surface due to the deposition process.

During the atomic force microscopy analysis, the deposition of materials was verified, both on the electrode surface (the region between the electrodes) and in the digits (Figure 5). This material comes from the use of electrodes in samples of pseudo-binary mixtures of biodiesel–diesel. The geometry of the interdigitated electrodes favored the deposition of nanostructures between the digits, since the dimensions were reduced. Furthermore, it was also possible to observe irregular edges on the electrodes, possibly related to the manufacturing process of the thin layers and mechanical stability. Other characteristics observed in the AFM height images are grooves in the region on the thin layer of gold deposited on the quartz substrate. These grooves can best be seen in three-dimensional images, as shown in Figure 5c. These scratches are associated with the method of depositing the gold layer on the substrate.

The AFM image of the interdigitated electrode shows that the conductive metal tracks were obtained uniformly along the dielectric substrate without major loss of contour.

**Figure 6:**
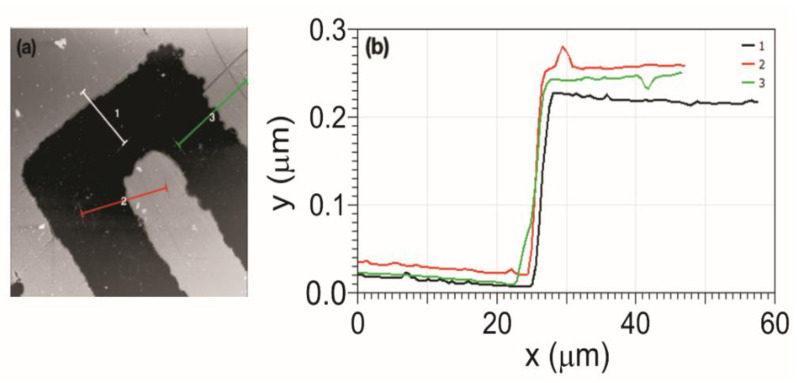
Cross-section analysis of the gold layer deposited on the quartz substrate. (**a**) Three regions have been selected, and their respective cross sections are shown in figure (**b**). The step height values are 225.4 nm (Region 1), 236.8 nm (Region 2), and 235.2 nm (Region 3).

Due to the AFM’s ability to acquire three-dimensional images, it was possible to measure the step height of the sensor’s gold layer. Several cross-sections were obtained, and the mean step value was 225.71 ± 0.0032 nm. Figure 6 shows examples of cross-sectional graphics in three different sensor regions. The steps shown in Figure 6b show a well-defined profile of the gold layer.

**Figure 7:**
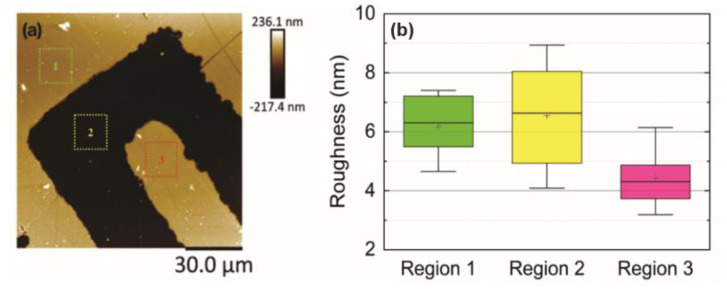
Roughness analysis obtained by Atomic Force Microscopy. (**a**) AFM topographic image indicating regions in which roughness analysis was performed. Each measure square has 10 µm^2^, and 12 measurements were acquired for each region. Region 1 (green square) corresponds to the sensor surface between the digits, Region 2 (yellow square) corresponds to the quartz substrate on which the gold layer was deposited, and Region 3 (red square) corresponds to the surface of the digit. (**b**) Boxplot graphs comparing the roughness values and average for each region. The mean values of quadratic roughness for each region were 6.17 ± 1.3 nm, 6.54 ± 1.9 nm, and 4.42 ± 1.0 nm for Region 1, Region 2 and Region 3, respectively.

From the AFM images, the mean square roughness of the sensor surface was obtained. Three regions were analysed: the gold layer on the digits, the gold layer on the interdigital region, and the quartz substrate, as illustrated in Figure 7. The area for calculating the roughness over all these regions was 10 µm^2^, and 12 squares were acquired for each region.

The roughness obtained for Region 1 was 6.17 ± 1.3 nm; for Region 2, it was about 6.54 ± 1.9 nm; and for Region 3, it was 4.42 ± 1.0 nm. This difference in roughness across the different regions of the sensor can be attributed to the following characteristics: (i) The roughness on the gold surface of the sensor, not corresponding to the digits, has a higher roughness when compared to the fingers due to the gold deposition process. In this region, the AFM images revealed several scratches (Figure 5b,c), increasing the roughness. (ii) In the quartz substrate, as shown in the three-dimensional image of the sensor (Figure 5c), several nanoparticulated residues were trapped there due to it being a region of depression in the topography, increasing its roughness value concerning the sensor body. (iii) In the finger region, the roughness is lower when compared to the other regions. This can be considered to be advantageous, as it is the most important region of the sensor. Although there are differences in the roughness, the whole sensor has nanometric roughness.

According to [31], surface roughness on solid electrodes alters the impedance response when coupled with the effect of nonuniform current distribution. However, for low roughness factors, the frequency dispersion is less significant than those caused by the geometric distribution of the electrodes. Thus, in this work, the effect of nanometric roughness of the electrode materials was neglected from the start.

According to [32], for applications in electronics, the interdigitated electrodes must have low surface roughness; considering the values obtained, it can be said that the surface of the interdigitated electrodes is suitable for this type of technological application. In addition, this surface can be modified with thin films of conductive materials (conductive polymers), thereby improving its sensitivity and even its selectivity to a certain analyte.

**Figure 8:**
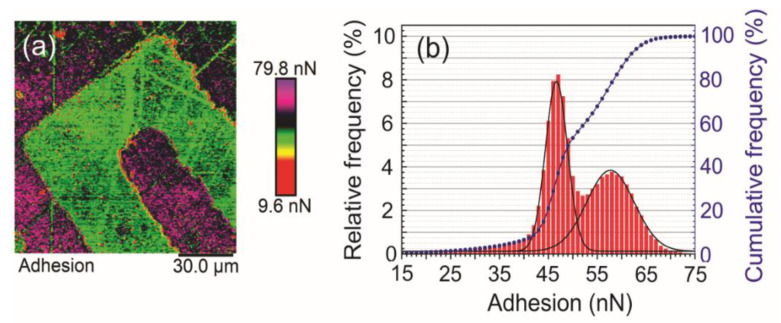
Adhesion analysis obtained by Atomic Force Microscopy. (**a**) AFM adhesion map over the sensor body. The map shows increased adhesion over the gold layer (purple) when compared with quartz substrate. (**b**) Adhesion values were plotted as a distribution and Gauss adjusted. Two distributions can be observed: one corresponding to quartz, with a value of around 46.6 nN of adhesion force, and the other one corresponding to the gold layer, with a Gauss distribution of around 57.7 nN. The cumulative frequency indicated two regimes, with the first one reaching saturation at approximately 50 nN and the second one at approximately 65 nN.

Another AFM investigation was the distribution of adhesion forces on the sensor surface. Figure 8a shows the AFM adhesion map on the sensor surface. The gold surface (purple) was greater than the adhesion on the quartz substrate (green). The adhesion values were plotted as a distribution (red) and Gauss adjusted (black curve). The first Gaussian corresponds to the adhesion values on the quartz and has a central value of 46.6 nN. The second Gaussian, corresponding to the gold film, has a value of 57.7 nN. The cumulative frequency graph (blue) shows two regimes, with the first achieving saturation (50%) at approximately 50 nN (quartz) and the second at 65 nN (gold) (100%).

Regarding to the physical stability of the surface, no major degradation of the electrode surface was observed due to corrosion of the metallic layer or equivalent decomposition of the dielectric substrate. However, the atomic force micrographs showed that the physical cleaning using ethanol and soft paper could generate scratches on the electrode surface (Figure 8). This drawback was solved by using 2-propanol as a cleaning solvent under NTP conditions.

**Figure 9:**
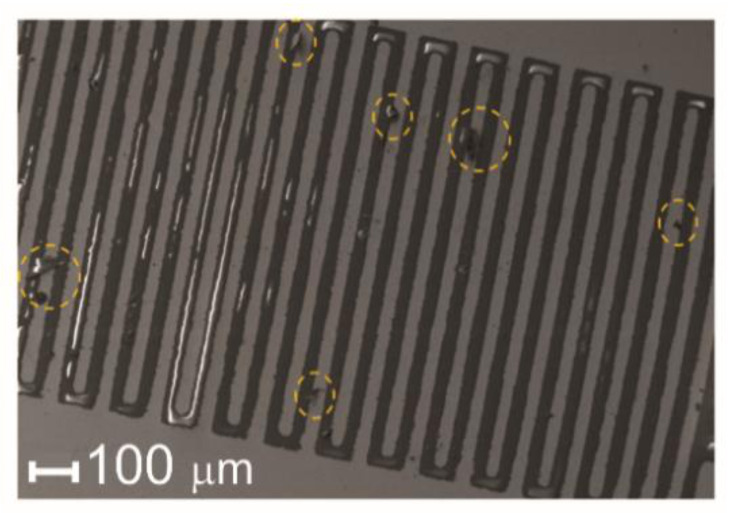
Scanning electron microscopy image of the electrode digit region. The yellow circles show small gaps in the conductive layer.

Scanning electron microscopy was used for evaluating the quality of the manufactured electrodes. In general, all the produced electrodes showed small surface irregularities, probably due to imperfections caused by the microelectronic photolithography method. Figure 9 shows regions (yellow circles) where both metal deposition and metal discontinuity were observed; however, no other extensive cracks or factures were detected. These characteristics guarantee a better distribution of charges in electrodes, allowing a large area of interaction with the analyte.

According to [33], to apply interdigitated electrodes as sensors, biosensors, and even electronic tongue and nose systems, metal electrodes are needed to display precise patterns on their surfaces, obtained by removing metallic layers, thereby producing the digits. These results indicate that the electrodes obtained by this method were appropriate for the fabrication of structured devices suitable for biosensors and molecular electronics. However, using lasers with a high repetition rate is an efficient approach to achieve these characteristics and a viable solution for the microelectronic photolithography method, which is a time-consuming and expensive method.

### 3.3. Electrical Characterization

#### Sensitivity of Electrodes to Different Fuels

**Figure 10:**
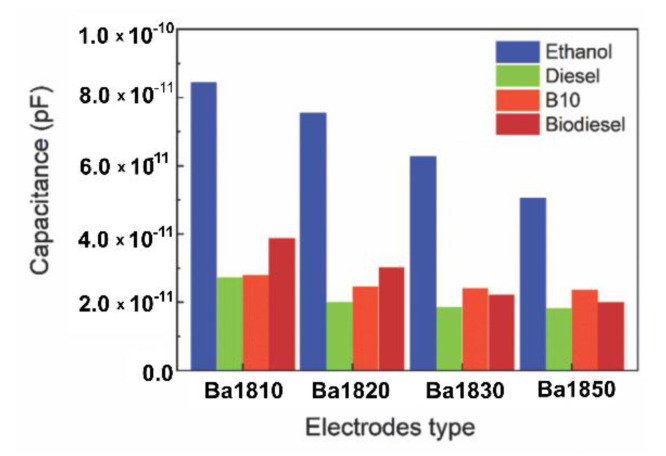
Response of different microelectrode geometries for fuel samples at 1000 Hz and 0.2 V.

Electrodes differentiated by the spacing between digits, as shown in Table 1, were immersed in samples of ethanol, petrol, diesel, and biodiesel to study their sensitivity. In Figure 10, we can see the capacitance response of the four electrodes differentiated by the spacing between the digits, and we found that the Ba1810 sensor obtained the highest capacitive value for the fuel samples. However, the capacitive detection of the interdigitated electrode is based on the following equation of permittivity and the relative permittivity of a dielectric material capacitance:

(5) $$\;C=\frac{\varepsilon_{0}\varepsilon_{r}A}{d}\;$$

where $\varepsilon_{0}$ and $\varepsilon_{r}$ represent the vacuum between electrodes, respectively, and d and A represent the electrode distance and area, respectively [34]. Observing the capacitance equation, we can deduce that the smaller the area between the digits, the greater the capacitance of the system; having a small distance between the combs maximizes the value. This predisposition was also observed in the simulations (Figure 2c).

Also, the electrodes showed a greater response to ethanol compared to other fuels. As mentioned earlier, this behavior can be attributed to the dielectric constants of biodiesel, diesel, and petrol being lower than the dielectric constant of ethanol [35]. It is also interesting to note that the value of the capacitance of the sensor depends on the geometric capacitance between the interdigitated electrodes, the contribution of the glass substrate, and the liquid. According to [36], when the built sensor’s capacitive trails are close enough, the field lines act on the entire surface of the sensor, increasing the capacitance of the device.

The edge field effect may be associated with the values obtained in Figure 10. It was observed that there is an influence of the electric field in the material that was tested, in this case, ethanol, diesel, B10 mixture, and biodiesel. This interaction occurs mainly at the edges of the capacitor. Thus, the distance between the sensor digits and its angle influences the electric field and, consequently, the capacitance of the system [37].

It is important to note that although the apparent capacitance of organic pseudo-binary mixtures can be altered by a non-uniform electric field, as predicted by [38], such an effect was not detected in our experiments, probably due to the chemical equivalences of higher molecular weight hydrocarbons and alkyl ester of fatty acids, both pristine constituents of the analytes.

To understand the phenomena that occur at the electrode interfaces, impedance spectroscopy measurements were performed. This technique is very useful for the characterization of the electrical behavior of solid or liquid materials (ionic, semiconductor, and dielectric) and electronic devices [39].

**Figure 11:**
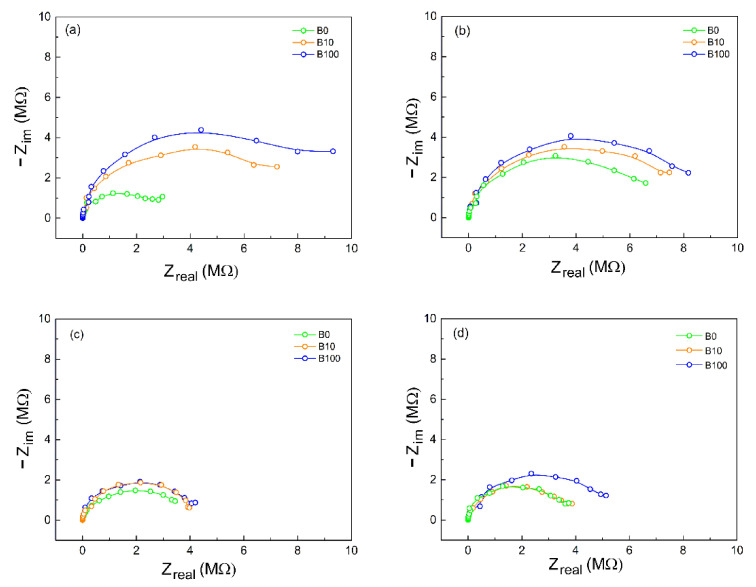
Nyquist diagrams of the fuel samples, using the electrodes: (**a**) Ba1810, (**b**) Ba1820, (**c**) Ba1830, and (**d**) Ba1850.

Such an experiment aims to differentiate the electrical properties of these media, envisioning the proposition of a method to determine the content of biodiesel and possible contaminants. In this context, the electrical behavior was determined qualitatively for the samples of biodiesel (B100), diesel S10A (B0), and diesel S10B (B10) under the experimental conditions described. According to Figure 11a and b, both Ba1810 and Ba1820 electrodes showed, a priori, a higher sensitivity towards the variation of complex impedance as a function of the biodiesel content in the analytes, indicating a significant responsiveness of these probes to the charge transfer process [40].

The spectra consist of well-defined semicircles, each of which could be ideally assembled using an equivalent circuit consisting of a parallel resistance–capacitance network. The semicircle corresponds to the process of electron transfer at high frequencies. On the left side of the semicircle, the impedance of the system is dominated by the resistance of the solution (R_s_). On the right side, in the region of low frequencies, the impedance of the system is dominated by the sum of the solution resistance and the charge transfer resistance (R_ct_) [41,42]. According to [43], the R_ct_ is mainly affected by the kinetics of electron transfer from the electrolyte to the metal and depends on the concentration of species, temperature, applied potential, and the structure of the interface.

As shown in Figure 11, the impedance values of biodiesel, diesel, and the B10 mixture are in mega-ohms, indicating a very high electrical resistance of the liquid. According to [44,45], these values align with the expected values for viscous and non-polar liquids. Similar impedance values of giga-ohms and even tera-ohms have been observed for biodiesel fuels and mixtures [46].

In Figure 11, in the region of low frequencies, we can see more clearly that the tendency of forming a second capacitive arc is more evident in the samples of B100 than for the media where there is the presence of diesel, indicating a possible interaction of biodiesel with the surface of the electrode. This fact was also observed by [47]. Furthermore, the value of charge transfer resistance for B0 is lower when compared to B10 (which has 10% biodiesel) and B100, demonstrating, in principle, that the first is a more conductive medium when compared with the last two.

Moreover, Figure 11 suggests that the addition of biodiesel increases the conduction processes in biodiesel–diesel mixtures. According to [48], monitoring the dielectric constant of the materials (calculated from capacitance) is more promising to assess the biodiesel content in mixtures of biodiesel–diesel than the use of resistivity measures.

**Figure 12:**
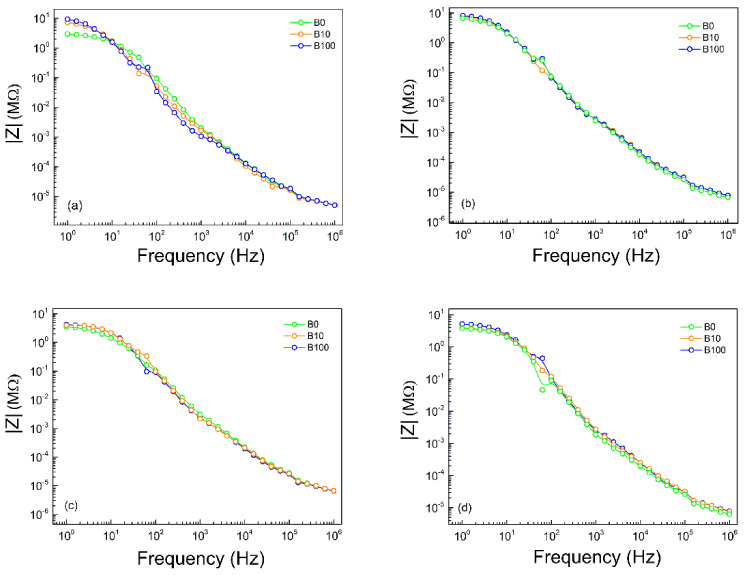
Bode diagram of the fuel samples, using the electrodes: (**a**) Ba1810, (**b**) Ba1820, (**c**) Ba1830 and (**d**) Ba1850.

As shown in Figure 12, the impedance of the fuel sample declined with increasing signal frequency, especially in the range higher than 100 Hz. However, in the high-frequency region, the differences in the values of the impedimetric signals between the fuels with different blending ratios are not significant. Nevertheless, in the low-frequency region from 1 to 100 Hz, there are apparent impedimetric differences in discriminating and estimating the blending ratio of biodiesel.

Analyzing the Bode diagram, Figure 12 shows that the addition of a fixed volume of biodiesel in the cell causes a shift in the impedance value in the low-frequency region to even greater values. According to [49], biodiesel may be interacting with the metallic surface, thereby contributing to the formation of this insulating film and thus hampering the passage of electric current at the interface. This behavior can also be observed in diesel S10A. It is believed that such behavior may be related to accumulations of organic species present in this medium and the presence of sulphur [50].

According to [51], the modulus of impedance does not change with the magnitude of the impedance, indicating resistive behavior. The values of real and imaginary components are in the order of MΩ cm^−2^, and a good correlation between experimental and simulated data was obtained.

**Figure 13:**
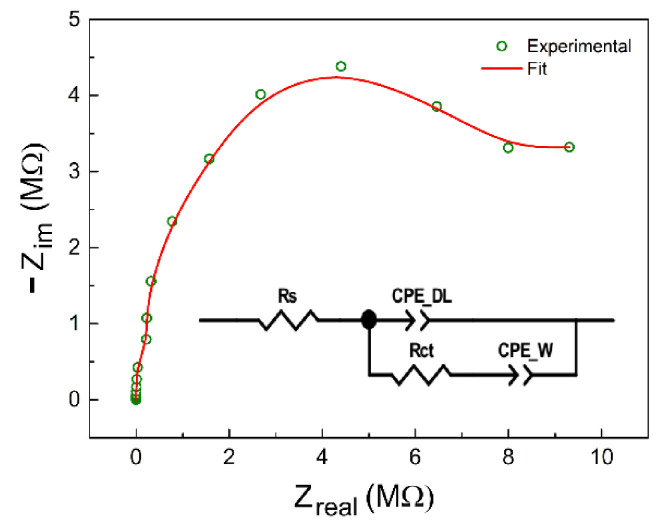
Adjustment of the proposed equivalent circuit for the pseudo-binary mixture of biodiesel–diesel.

Figure 13 shows an equivalent circuit for the studied systems proposed to interpret the results shown above. The circuit is formed by a resistor (R_s_) in series with a parallel arrangement of a constant phase element (CPE_DL_) with the resistance R_ct_ that is in series with the constant phase element CPE_W_. We can observe capacitance at low frequencies, represented by a constant phase element (CPE_DL_) in the circuit, possibly due to a passive layer or a non-conductive film adsorbed on the metal surface that prevents charge transfer. The values for each parameter can be seen in Table 2. All components are verified by the chi-square evaluation, with a 95% confidence level.

The constant phase element (CPE) resembles a capacitor and a resistor depending on the intrinsic values of this component. That is, the CPE is used when the thermodynamic analysis of the analytes presents non-linearity in the diffusion of charges through the solution [52]. The component CPE_W_ acts in the low-frequency region as a Warburg impedance.

The constant phase element consists of two constants, expressed by

(6) $$Z_{CPE}=\;\frac{1}{T{\left(j\varpi \right)}^{P}}$$

The component P (CPE-P) varies from 0 to 1. When the value is equal to 1, this component presents a characteristic of a capacitor (greater effect of the electronic double layer); when it is equal to 0, the element tends to be a resistor. When *P* = 0.5, the CPE-P becomes a Warburg impedance [53].

We also found that the charge transfer resistance (R_ct_) decreases linearly with the amount of biodiesel in the analyte, suggesting a meaningful responsiveness of the designed electrodes to charge transfer phenomena. A similar result was also observed by [54].

## 4. Discussion

This investigation associates the capacitance values obtained in the simulations to define the best architecture of interdigitated electrodes, in addition to the characterizations (morphological, structural, and electrical) of the manufactured electrodes. The performance of a capacitive sensor, signal strength, depth of penetration, and metrological sensitivity is mainly connected to sensor geometry. A 3D electrostatic simulation based on finite element methods was used to determine the topology of interdigitated electrodes to be manufactured, since the ability to measure analytes depends on the topology of the electrode. Based on the modeling performed, it can be assumed that the geometric parameters more suitable for the construction of the electrode are the following: W = 20 µm, L = 1000 µm, G = 50 µm, and N = 40 digits.

Furthermore, it was possible to demonstrate the effects of the electric field between the electrode digits. It was observed that under certain conditions, the magnitude of the electric field is greater among the electrodes with narrower spacing between the digits, Figure 3. According to [55], the magnitude of the electric field above the interdigitated electrodes is proportional to the spacing between the digits. Also, it depends on changes in the dielectric properties of the materials.

Also, in Figure 3, we can observe the electric field’s penetration depth, which can be defined as the maximum distance in the z-direction for the sensor to produce a detectable change in the sensor output (capacitive planar sensor). According to [56], it is an important parameter to indicate how far the sensor can be sensitive, being determined by the permissiveness of the material and the geometry of the electrode. In this context, we observe in Figure 3 that although the electrode with the largest conductive layer has a greater capacitance than the one with the lowest conductive layer, the depth of penetration of the electric field is practically the same.

Regarding the structural characterization by X-ray diffraction (Figure 4), it was possible to observe peaks of diffraction resembling two distinct phases on the surface of the electrodes: Au^0^ (conductor) and SiO_2_ (dielectric substrate). It is not possible to verify the added chromium layer to promote greater adherence of gold to the substrate.

Concerning the depth of the devices in Figure 5 and Figure 6, it was possible to observe that the electrodes have a gold conductive layer of 225.71 ± 0.0032 nm. Although differences in roughness have been observed due to the gold deposition process, scratches in the conductive layer, and nanoparticle residues, the entire sensor has nanometric roughness (Figure 7).

The distribution of adhesion forces on the sensor surface was also assessed (Figure 8). This study is important because fuel molecules must attach to the surface of the sensor—no problems for eventual surface modification were found, which promoted greater sensitivity to the analyte of interest.

Through scanning electron microscopy of the electrodes (Figure 9), it was possible to verify some irregularities on the surface and edges attributed to the microelectronic photolithography method. These imperfections could be corrected using the laser ablation method.

The sensitivity of the interdigitated electrodes was also investigated, showing that the manufactured devices with narrower spacing between the combs (Ba1810 and Ba1820) demonstrated greater sensitivity to fuels, as can be seen in Figure 10. Furthermore, the electrodes showed a greater response to ethanol than other fuels. As mentioned earlier, this behavior can be attributed to the dielectric constants of biodiesel, diesel, and gasoline being lower than the dielectric constant of ethanol.

The electrical behavior observed in the Nyquist diagram of Figure 11 is characterized by a capacitive arc and, in the low-frequency region, another behavior that can be attributed to interfacial phenomena [57,58]. It was also possible to observe a loss of sensitivity in medium- and high-frequency regions, making it possible to distinguish between fuels in the low-frequency region, as shown in Figure 12.

In the equivalent circuit shown in Figure 13, we observe that the resistance R_s_ comes from the species present in the pseudo-binary biodiesel–diesel mixture; that is, it is the resistance of the solution. The metal interface with the mixture is represented by the resistance R_ct_, corresponding to a charge transfer resistance. The double-layer capacitance is represented by CPE_DL_ and CPE_W_, related to the insulating film formed on the surface of this type of electrode. This reinforces the hypothesis of forming a capacitive film on the metal surface of the electrode. The method is reliable and indicates good analytical performance because it presented small errors between the experimental and simulated data (Table 2).

After studying the electrical properties, we found that the value of the charge transfer resistance (R_ct_) decreases with the addition of biodiesel into the system. In this context, we can determine that the value of R_ct_ depends on the presence of biodiesel in the system. Future work should focus on further optimization of conditions to maximize results and develop a device that can give an, estimate of the quality of the fuel.

## 5. Conclusions

In this study, a sensory device based on interdigitated electrodes to determine additives in biodiesel and diesel mixtures is proposed. To this end, simulations were used to determine the topology and ideal conditions for using interdigitated electrodes as fuel sensors. The electrical characterization results show that it is possible to differentiate the fuels and determine the biodiesel content in the diesel in the low-frequency region by using the developed electrodes. The analytical method also provides data that could be used in other applications, such as assessing the contamination or purity of biodiesel. It is worth mentioning that the proposed method has a lower cost when compared to the official methodology adopted by the National Agency of Petroleum, Natural gas and Biofuels (ANP) (EN 14078 and NBR 15568). Moreover, the electrodes can be used as an alternative method to evaluate the quality of biodiesel–diesel mixtures and other pseudo-binary liquid fuel mixture compositions.

## Figures and Tables

**Table 1:** The physical dimensions of the interdigitated electrodes.

Sensor Coding	Comb Spacing (µm)	Effective Area (mm²)	Number of Electrodes (Units)
Ba1810	10	1.25	20
Ba1820	20	1.65	20
Ba1830	30	2.00	20
Ba1850	50	2.80	20

**Table 2:** Values obtained after adjustments made based on the proposed equivalent electrical circuit for the B0, B10, and B100 analytes (Ba1810 electrode).

Circuit Element	B0	B10	B100
R_s_	5421	3984	3530
R_ct_	5.08 × 10^8^	4.02 × 10^8^	3.16 × 10^8^
CPE_DL_—T	3.52 × 10^−12^	3.56 × 10^−12^	3.59 × 10^−12^
CPE_DL_—p	0.97	0.97	0.98
CPE_W_—T	5.33 × 10^−10^	5.40 × 10^−10^	5.44 × 10^−10^
CPE_W_—P	0.49	0.46	0.45
$\chi^{2}$	0.003	0.007	0.005
